# Three-dimensional dose comparison of flattening filter (FF) and flattening filter-free (FFF) radiation therapy by using NIPAM gel dosimetry

**DOI:** 10.1371/journal.pone.0212546

**Published:** 2019-02-21

**Authors:** Chun-Hsu Yao, Tung-Hao Chang, Chia-Chi Lin, Yuan-Chun Lai, Chin-Hsing Chen, Yuan-Jen Chang

**Affiliations:** 1 Biomaterials Translational Research Center, China Medical University Hospital, Taichung City, Taiwan, R.O.C.; 2 Department of Biomedical Imaging and Radiological Science, China Medical University, Taichung City, Taiwan, R.O.C.; 3 School of Chinese Medicine, China Medical University, Taichung City, Taiwan, R.O.C.; 4 Department of Biomedical Informatics, Asia University, Taichung City, Taiwan, R.O.C.; 5 Department of Radiation Oncology, Changhua Christian Hospital, Changhua City, Taiwan, R.O.C.; 6 Department of Management Information Systems, Central Taiwan University of Science and Technology, Taichung City, Taiwan, R.O.C.; 7 Department of Aerospace and Systems Engineering, Feng-Chia University, Taichung City, Taiwan, R.O.C.; Mar Ephraem College of Engineering & Technology, INDIA

## Abstract

Intensity-modulated radiotherapy and volumetric modulated arc therapy are modern radiation therapy technologies that can create the desired dose distribution by multileaf collimator movement and dose-rate control. However, the homogeneous dose delivery of small-field irradiation techniques shows disagreement with that of treatment planning system. The removal of the flattening filter by flattening filter free (FFF) beam irradiation increases dose conformity and further reduces treatment delivery time in radiosurgery. This study aims to investigate the dose distribution of FFF and flattened beams for small-field irradiation by using the 3D gel dosimeter. The *N*-isopropylacrylamide (NIPAM) polymer gel dosimeter was employed to record the 3D dose distribution. In addition, flattened and FFF beams were compared using the gamma evaluation technique. The use of an FFF accelerator resulted in excellent radiation treatments with short delivery times and low doses to normal tissues and organs. Results also showed that the passing rate increased with the decrease of field size (30 × 30, 20 × 20, and 10 × 10 mm^2^) at post-irradiation times of 24, 48, 72, and 96 h. The passing rates for each field size were retained at the same level when gamma criteria, namely, distance-to-agreement (DTA) = 3 mm/dose difference (DD) = 3%, were used. Nevertheless, the passing rates for each field size decreased slowly after 48 h when DTA = 2 mm/DD = 2%. The Wilcoxon signed-rank test was used to determine statistical difference with a significant level of *p* < 0.05. The passing rates of flattened and FFF beams showed no significant difference. The edge enhancement effect in the flattened beam was more evident than in the FFF beam. The 3D NIPAM gel dosimeter can be used for dose verification of small field for radiation therapy with high dose rate.

## Introduction

The introduction of stereotactic body radiotherapy (SBRT) can provide acceptable control probabilities to patients [1]. This technique is a high-precision radiotherapy used for treating small to moderate extracranial tumors [2]. The SBRT may deliver a greater biologic dose of radiation, thus it can shorten the treatment period. Intensity-modulated radiotherapy (IMRT) and volumetric-modulated arc therapy (VMAT), as modern radiation therapy technologies, can create the desired dose distribution by multileaf collimator (MLC) movement and dose-rate control. Vassiliev et al. [3] revealed that the FFF photon beam can reduce the out-of-field dose, because the removal of flattened filter can reduce the head scatter and softer spectra. Cashmore [4] also concluded similar results; the head scatter effect can be reduced approximately by 70% and reduction of out-of-field dose by 11% for a 6 MV FFF photon beam. Peguret et al. [5] adopted FFF beam for lung treatment and found that the treatment time was significantly reduced. Stieler et al. [6] used 6 MV standard vs. 6 MV FFF mode radiation beams for brain metastasis treatment. Their results showed that FFF mode treatment can reduce the treatment time to 51.5% with similar MU for VMAT and IMRT radiation. The gamma evaluation can achieve 99.08 ± 1.58% and 93.46 ± 2.41% with gamma criteria 3% dose difference (DD)/3 mm dose to agreement (DTA) and 3% DD/1 mm DTA, respectively. Navarria et al. [7] performed a clinical study to assess the outcome between 3DCRT with flattened beams (FF) and VMAT with FFF beams for SBRT. Both radiation modes can achieve dose conformity. Moreover, VMAT with FFF mode showed statistically significant reduction of ipsilateral lung doses and beam-on-time. In addition, local control rate was as high as 100% with FFF mode but was only 92.5% with FF beams (*p* = 0.03) at 1-year follow-up.

A region with a high dose gradient appears in the edge of radiation field because of the thin penumbra for the FFF mode. To obtain a true 3D dose distribution of FFF mode radiation, the gel dosimeter is a good choice. Olding et al. [8] adopted a 1D ion chamber, a 2D Gafchromic EBT3 film, and a 3D gel dosimeter to validate the two radiation modes of VMAT stereotactic ablative radiotherapy for a motion phantom. Both measurement dose distributions were consistent. However, this distribution also showed that the most disagreement of dose profile was located at the edge of high dose region. Pavoni et al. [9] proposed a composite gel-alanine phantom to validate a VMAT treatment of multiple brain metastases. The results were consistent with those of planning data. McErlean et al. [10] established a new 3D dosimetry combined with optical computed tomography (optical CT) to perform the dose verification of synchrotron microbeam radiation therapy (MRT) dose distribution. The optical CT and gel dosimeter can provide an excellent MRT visualization. The result can be useful for identifying potential treatment anomalies. Waldenberg et al. [11] adopted the NIPAM gel dosimeter combined with magnetic resonance imaging to investigate the dose dependence of the gel dosimeter. An important observation from their result was that the dose response of the gel is dependent on sequential beam irradiation. In other words, a high dose rate gives a low dose response. However, the sequential irradiation dependence is less pronounced if the time between the sequential beams had been reduced. Yao et al. [12] showed that the treatment planning system (TPS) map can achieve higher conformity to the planning target volume than that of IMRT. Three-dimensional (3D) gel dosimetry was used to validate the dose distribution agreement of VMAT, and the results showed good performance [12]. The passing rate can be remarkably high at approximately 95% agreement compared with that of TPS, with 3%/3 mm criterion [13,14]. The dose verification of single square-field and multifield irradiation of IMRT was also performed by the 3D gel dosimeters [15]. Nevertheless, the homogeneous dose delivery revealed disagreement with TPS when using small-field irradiation techniques [16,17]. The flattening filter free (FFF) beam irradiation increased the dose conformity and further reduced the treatment delivery time in radiosurgery by removing the flattening filter [1,18,19]. When performing stereotactic ablative body radiotherapy, in which the intrafraction organ/patient motion may affect the delivery efficacy, treatment time may be an important issue. Therefore, this study aims to investigate the dose distribution of FFF and flattened beams for small-field irradiation by using a 3D gel dosimeter. FFF and flattened beams were also compared using gamma evaluation. We utilized a simple square field irradiation in the current study because of the sequential irradiation dependence.

## Materials and methods

### Gel preparation

*N*-isopropylacrylamide (NIPAM) polymer gel was used in the current study. The NIPAM polymer gel dosimeters were prepared by a method similar to that described by Senden et al. [20]. Nonetheless, the original formulation was modified to obtain high sensitivity and linear gel recipe [21]. As shown in Table 1, the gel recipe includes 5% gelatin (300 Bloom Type A, Sigma-Aldrich, St. Louis, MO, USA), 5% NIPAM (97% pure; Wako, Osaka, Japan), 3% *N*,*N′*-methylene bisacrylamide (Bis, Sigma-Aldrich, St. Louis, MO, USA), and 5 mM tetrakis (hydroxymethyl) phosphonium chloride (THPC, TCI, Sigma-Aldrich, St. Louis, MO, USA). In the beginning, the gel phantom was heated to 45°C gradually in a water bath and stirred continously until the gelation solution became clear and transparent. Then, 3 wt% Bis was added and dissolved at 45°C by continuous stirring for 15 min. Subsequently, 5 wt% monomer was added and dissolved at 45°C and kept stirring for approximately 15 min. Finally, 5 mM THPC was added to the solution and dissolved at 42°C by continuous stirring for 2 min until the resulting gel finally became clear and transparent. The acrylic phantom with the dimension of 10 cm in diameter, 10 cm in height, and 3 mm in wall thickness, was filled with gel. Then, the gel phantom was immersed in a refrigerator with a fixed temperature of 6°C for at least 6 h until complete solidification. Further details on the gel preparation are provided in the work of Hsieh et al. [22]. To avoid the oxygen effect on the gel polymerization, the acrylic phantom cap was sealed with parafilm to prevent oxygen from entering into the phantom. The entire gel phantom was wrapped entirely by an aluminum foil to prevent photopolymerization.

**Table 1:** Gel recipe used in this study [11].

Composition	Weight (%)	Amount^a^
Gelatin	5	5±0.001 g
*N*-isopropylacrylamide (NIPAM)	5	5±0.001 g
*N*,*Nʹ*-methylene bisacrylamide	3	3±0.001 g
Tetrakis (hydroxymethyl) phosphonium chloride	5	8.96×10^−2^±1×10^−3^ mL
Distilled water	87	87±0.1 mL

^a^The composition was determined according to the preparation of 100 mL NIPAM dosimeter.

### Irradiation and TPS

This study adopted Elekta Versa HD linear accelerator (Elekta AB, Sweden), which was provided by Changhua Christian Hospital. This accelerator provided two modes of irradiation including the flattened photon beam mode and the FFF photon beam mode. The gel phantom was aligned to the center line of the treatment head. The pinnacle TPS (Pinnacle3 version 9.8, Philips, USA) was used. The pinnacle TPS for small-field irradiation was calibration by using a beam data obtained from small field 10 × 10 mm2 and 20 × 20 mm2 radiation, which was measured by a pinpoint ion chamber (0.015c.c)(PinPoint Ionization Chambers 30014, PTW, Germany). The target dose was 5 Gy at 5 cm depth of the NIPAM gel dosimeter. The source-to-surface distance was 95 cm, and the gantry angle was 0°. The dose rate in the FF mode was 600 MU/min, with 6 MV photon energy. In the FFF mode, the dose rate was 1,400 MU/min, with 6 MV photon energy. The field sizes were 30 × 30, 20 × 20, and 10 × 10 mm^2^. The parameters of each batch are shown in Table 2. Eighteen gel dosimeters were prepared for three field sizes and two irradiation modes. Eighteen batches of gel phantoms were prepared.

**Table 2:** Parameters of each batch.

Irradiation mode	Flattened photon beam			Flattening filter free (FFF) photon beam		
Dose rate (MU/min)	600			1400		
**Field size (FS, mm**^**2**^**)**	30×30	20×20	10×10	30×30	20×20	10×10
**Field mode**	MLC	MLC	MLC	MLC	MLC	MLC
**Monitor unit (MU)**	562	587	653	547	573	625
**Time of beam on (s)**	56.2	58.7	65.3	23.4	24.6	27.9
**Batch**	1, 13, 15	3, 7, 11	5, 9, 17	2, 14, 16	4, 8, 12	6, 10, 18

### Gel phantom scanning and image reconstruction

**Fig 1:**
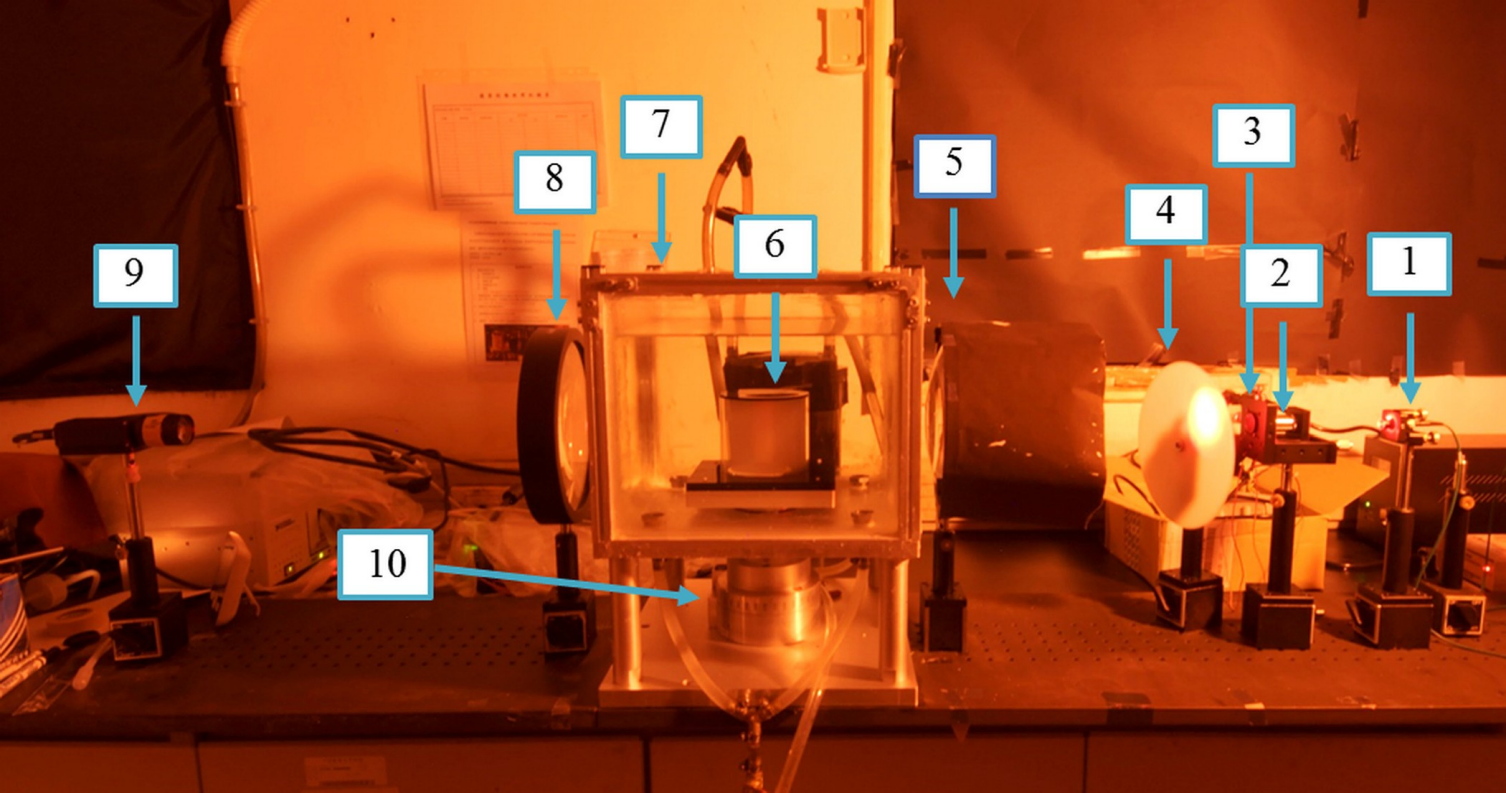
Image of the optical computed tomography scanner CT-s2. 1. laser, 2. spatial filter, 3. pinhole, 4. rotating diffuser, 5. collimating lens, 6. NIPAM gel, 7. aquarium, 8. collimating lens, 9. CCD camera, and 10. stepping motor.

We developed the proposed charge coupled device (CCD)-based parallel-beam optical-CT scanner (CT-s2) shown in Fig 1 [15,23]. The CT-s2 was used as dose readout tool after the polymer gel phantom was irradiated. The CT-s2 comprises (1) a laser, (2) spatial filter, (3) pinhole, (4) rotating diffuser, (5) collimating lens, (6) NIPAM gel phantom, (7) aquarium, (8) collimating lens, (9) CCD camera, and (10) stepping motor. The laser beam was converted into a plane light source by using a spatial filter, a microscope objective lens, and a pinhole. A significantly uniform parallel light source can be obtained by speckle reduction technique [24]. The gel phantom was mounted on the stepping-motor-driven rotating table immersed in an aquarium. The aquarium filled with matching liquid with best-fit refractive index of 1.346 was used to reduce the refraction and reflection effects at the interface between materials with different refractive indices [25]. Totally, 360 projection images were acquired by the CCD camera. Then, the image reconstruction was performed using a filtered back-projection algorithm with our proposed program written in MATLAB [15,23–25].

### Gamma evaluation

The quantitative gamma evaluation proposed by Low et al. [26,27] was used for performance analysis. Given that the acceptance criterion was varied in low- and high-gradient regions, direct comparison between the measured and calculated dose distribution values in various planes was unsuitable. In gamma evaluation, the measured and calculated doses are compared using a combination of a dose-difference (DD) criterion and a distance-to-agreement (DTA) criterion [26]. Point-by-point calculation was performed by comparing the dose distribution from the calculated TPS and that measured by the gel dosimeter. To perform a comparison, we used 2D level gamma calculation. After calculation, the gamma map and gamma passing rate can be obtained. In this study, two gamma criteria for gamma evaluation were used, namely, 3% DD/3 mm DTA and 2% DD/2 mm DTA [27]. The gamma passing rate was calculated in the percentage area with *γ* < 1.

## Results and discussion

### Spatial uniformity of the non-irradiated gel phantom

**Fig 2:**
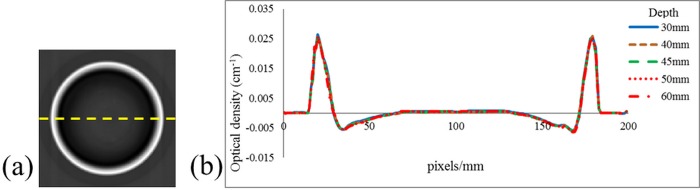
(a) Reference image of the gel phantom. (b) Line profiles of the gel phantom at depths of 30, 40, 45, 50, and 60 mm.

Differences in the temperature history experienced by a normoxic polymer gel formulation after its fabrication may influence the spatial uniformity of the gel phantom [28,29]. Fig 2 shows the scanning results of the reference image of the gel phantom (Fig 2A). The image is a reconstruction of a transverse slice. Fig 2B presents the line profiles of the scanning results of the non-irradiated gel phantom. These profiles illustrated the variations in the optical densities at different depths of 30, 40, 45, 50, and 60 mm. The maximum standard deviations between different depths in the central 60 pixel/mm region were less than 0.01%. The maximum standard deviation for all 18 batches was less than 0.3%. This result indicated high spatial uniformity for these batches of non-irradiated NIPAM gel dosimeters.

### Comparison of gamma passing rates between various irradiation modes

A quantitative gamma evaluation was performed between the dose map calculated from TPS and that measured by an optical-CT scanner. Table 3 shows the average gamma passing rate and standard deviation with gamma criteria of DTA = 3 mm/DD = 3% for various irradiation modes and FS at various depths and different post-irradiation periods. At FS = 30 × 30 mm^2^ and post-irradiation time of 24 h, 12 gamma passing rates corresponding to three batches of the NIPAM gel dosimeter and four depths at 30, 40, 50, and 60 mm were calculated. The means and standard deviations of flattened and FFF beams at post-irradiation time of 24 h were 97.41 ± 1.58 and 97.49 ± 1.95, respectively. The gamma passing rates were almost the same for the two types of irradiation mode. At FS = 30′30 mm^2^ and post-irradiation times of 48, 72, and 96 h, the means and standard deviations of gamma passing rates for flattened and FFF beams were 95.44 ± 2.25 and 96.30 ± 2.43, 96.77 ± 2.42 and 95.95 ± 2.12, and 96.31 ± 2.26 and 96.75 ± 2.47, respectively. The overall means and standard deviations of gamma passing rates for flattened and FFF beams were 96.48 ± 2.29 and 96.62 ± 2.35, respectively. We used the Wilcoxon signed-rank test to determine whether or not the passing rates of flattened and FFF beams show significant statistical difference. This non-parametric statistical hypothesis test is used to compare two related samples, matched samples, or repeated measurements on a single sample to assess differences in their population mean ranks. The Wilcoxon signed-rank test can be used as an alternative to the paired t-test, t-test for matched pairs, or the t-test for dependent samples when the population is assumed to be not normally distributed [30]. *p* < 0.05 was considered statistically significant. The passing rates of flattened and FFF beams were not significantly different (*p* = 0.566). The corresponding *p*-values when FS were 20 × 20 and 10 × 10 mm^2^ are shown in Table 3. The same conclusion can be obtained for two irradiation modes. Previous studies [31,32] reported similar findings and revealed that the dosimetric data show no statistically significant difference (*p* > 0.05) for the target coverage of flattened and FFF beams at 6 MV energy.

**Table 3:** Average gamma passing rate and standard deviation of various irradiation modes and FS at different post-irradiation periods with distance-to-agreement (DTA) of 3 mm and dose distance (DD) of 3%.

Post-irradiation time	24 h		48 h		72 h		96 h		Overall mean ±SD		Wilcoxon Test (*p*-value*)
Irradiation mode	**FS = 30**×**30 mm**^**2**^										
Flattened photon beam	97.41	±1.58	95.44	±2.25	96.77	±2.42	96.31	±2.26	96.48	±2.29	0.566
FFF photon beam	97.49	±1.95	96.30	±2.43	95.95	±2.12	96.75	±2.47	96.62	±2.35	
Irradiation mode	**FS = 20**×**20 mm**^**2**^										
Flattened photon beam	98.40	±1.24	98.45	±1.69	97.32	±1.86	97.69	±2.30	97.97	±1.90	0.622
FFF photon beam	98.01	±1.64	98.15	±1.15	97.34	±1.11	98.07	±0.87	97.89	±1.28	
Irradiation mode	**FS = 10**×**10 mm**^**2**^										
Flattened photon beam	100	±0.0	100	±0.0	100	±0.0	100	±0.0	100	±0.0	No difference exists.
FFF photon beam	100	±0.0	100	±0.0	100	±0.0	100	±0.0	100	±0.0	

*p > 0.05 represents no significant difference.

Gamma criteria of DTA = 2 mm/DD = 2% were used to calculate the passing rates, and the results are shown in Table 4. The passing rates with DTA = 2 mm/DD = 2% were lower than that with DTA = 3 mm/DD = 3%. The overall means and standard deviations of flattened and FFF beams at FS of 30 × 30, 20 × 20, and 10 × 10 mm^2^ were 85.11 ± 3.45 and 86.14 ± 4.85, 89.59 ± 4.11 and 88.88 ± 3.82, and 94.94 ± 2.12 and 94.73±2.14, respectively. When the FS was 30 × 30, 20 × 20, and 10 × 10 mm2, the corresponding p-values were 0.272, 0.406, and 0.626, respectively. For both gamma criteria DTA = 3 mm/DD = 3% and DTA = 2 mm/DD = 2%, the passing rates of flattened and FFF beams showed no significant difference (*p*-value > 0.05).

**Table 4:** Average gamma passing rate and standard deviation of various irradiation modes and FS at different post-irradiation periods with DTA = 2 mm/DD = 2%.

Post-irradiation time	24 h		48 h		72 h		96 h		Overall mean ±SD		Wilcoxon Test (*p*-value*)
Irradiation mode	**FS = 30**×**30 mm**^**2**^										
Flattened photon beam	85.18	±4.10	84.22	±3.46	83.85	±2.88	81.06	±2.61	85.11	±3.45	0.272
FFF photon beam	85.05	±4.83	85.30	±4.78	82.75	±4.64	84.47	±4.63	86.14	±4.85	
Irradiation mode	**FS = 20**×**20 mm**^**2**^										
Flattened photon beam	90.65	±2.50	90.68	±3.37	87.68	±4.61	88.32	±4.73	89.59	±4.11	0.406
FFF photon beam	89.54	±4.07	89.20	±3.04	88.17	±4.68	88.64	±2.91	88.88	±3.82	
Irradiation mode	**FS = 10**×**10 mm**^**2**^										
Flattened photon beam	95.76	±1.71	94.60	±2.21	94.82	±2.24	94.57	±1.96	94.94	±2.12	0.626
FFF photon beam	95.26	±2.05	94.99	±2.16	94.29	±2.27	94.41	±1.83	94.73	±2.14	

*p > 0.05 represents no significant difference.

### Comparison of temporal stability at various FS

**Fig 3:**
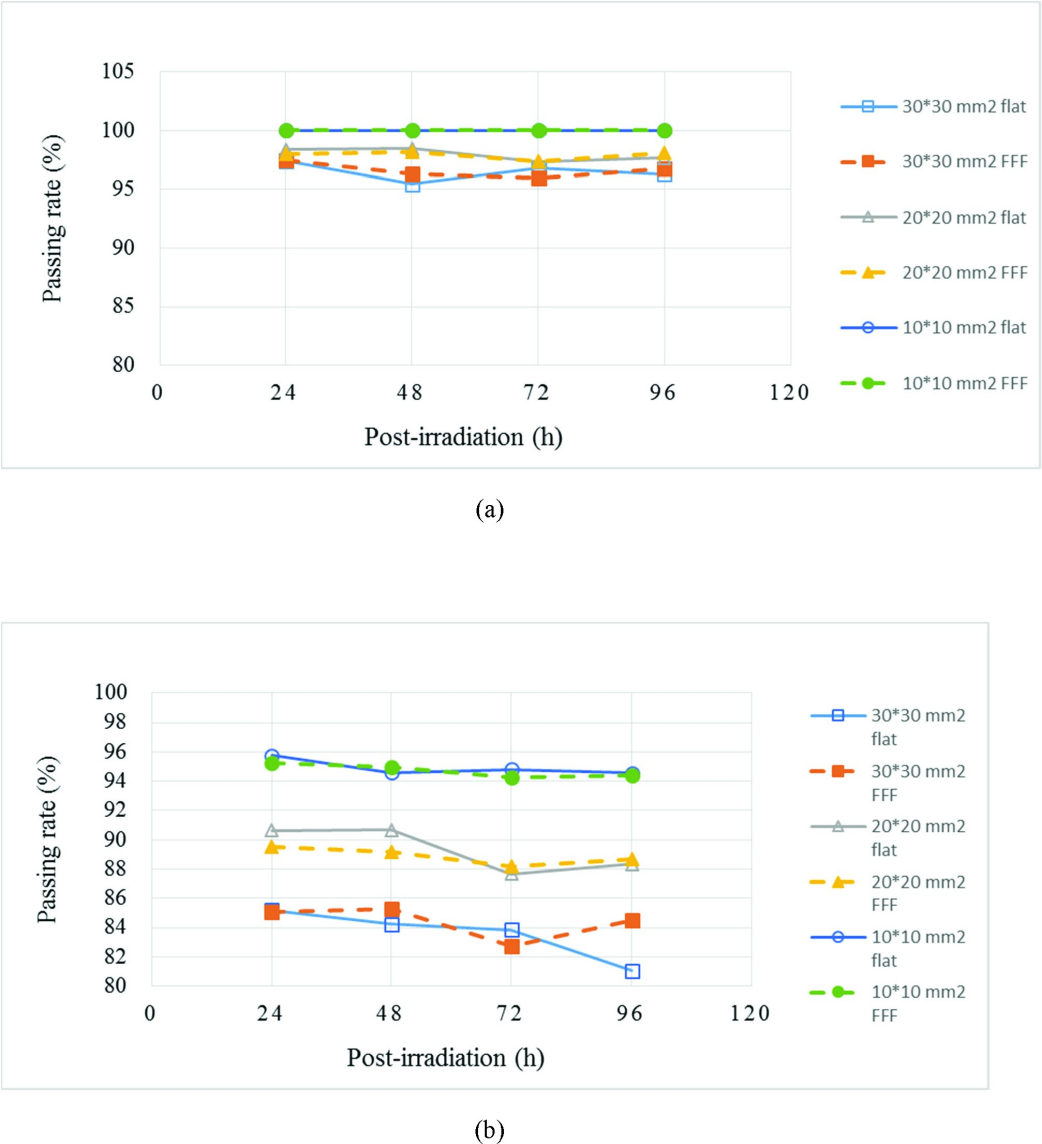
Gamma passing rate curve of various irradiation modes and FS at different post-irradiation periods with two gamma criteria: (a) DTA = 3 mm/DD = 3% and (b) DTA = 2 mm/DD = 2%. The solid line represents the flattened beam, and the dashed line represents the FFF beam.

As shown in Fig 3, the gamma passing rates of flattened and FFF beams at various post-irradiation periods and FS were plotted. The solid line represents the flattened beam, and the dashed line represents the FFF beam. The passing rate increased when the FS decreased from 30 × 30 mm^2^ to 10 × 10 mm^2^ at post-irradiation times of 24, 48, 72, and 96 h. For each FS, the passing rates retained the same level with DTA = 3 mm/DD = 3%. However, for each FS, the passing rates decreased slowly after 48 h when DTA = 2 mm/DD = 2%. Similar phenomenon was reported by Chang [16], who used an FS of 40 × 40 mm^2^.

### Comparison of gamma map between various irradiation modes and FS

**Fig 4:**
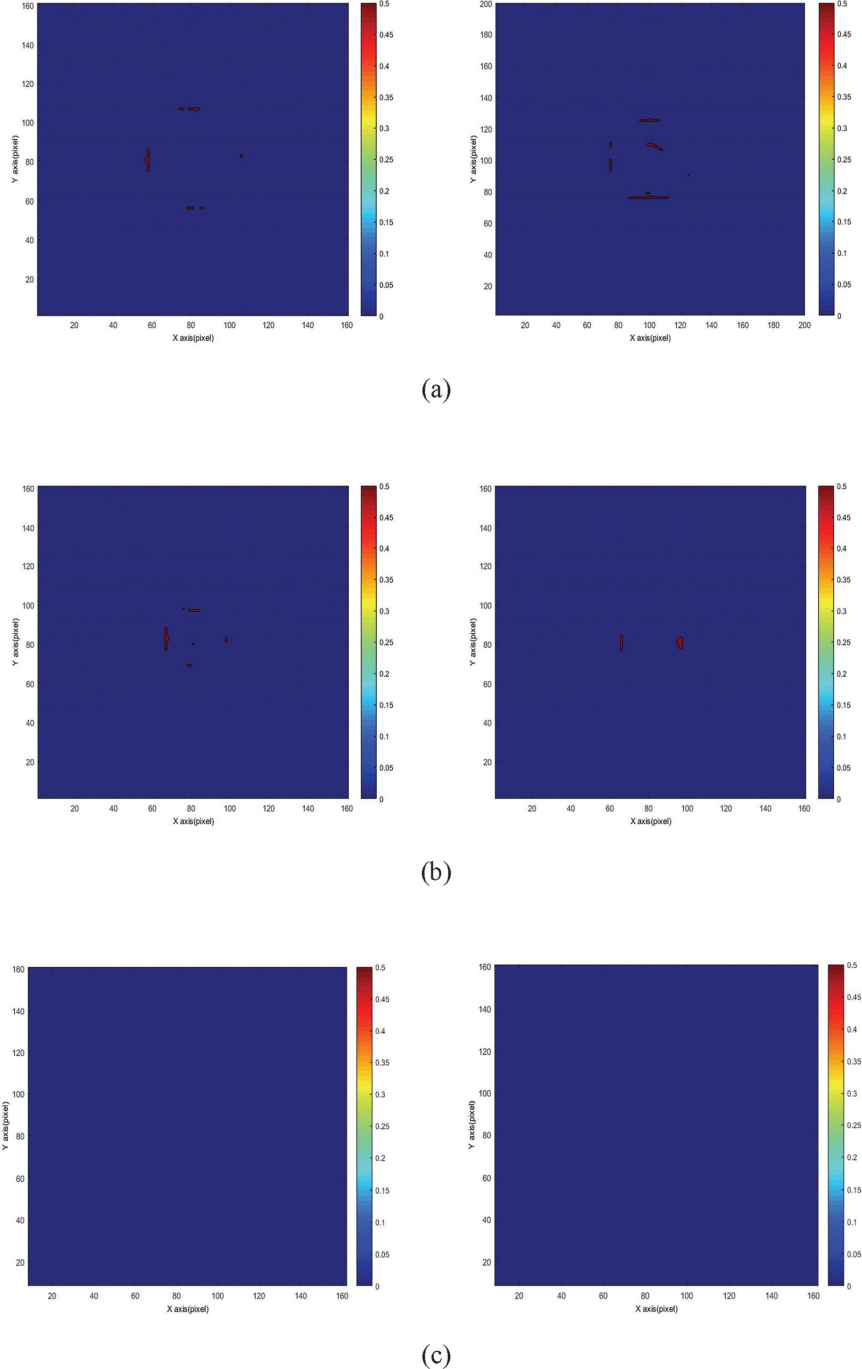
Various FS of the gamma map of the NIPAM gel dosimeter at a depth of 50 mm for 24 h post-irradiation time. The gamma criteria used were DTA = 3 mm/DD = 3%. The left image represents the flattened beam, and the right image represents the FFF beam. (a) FS = 30 × 30 mm^2^, (b) FS = 20 × 20 mm^2^, and (c) FS = 10 × 10 mm^2^.

**Fig 5:**
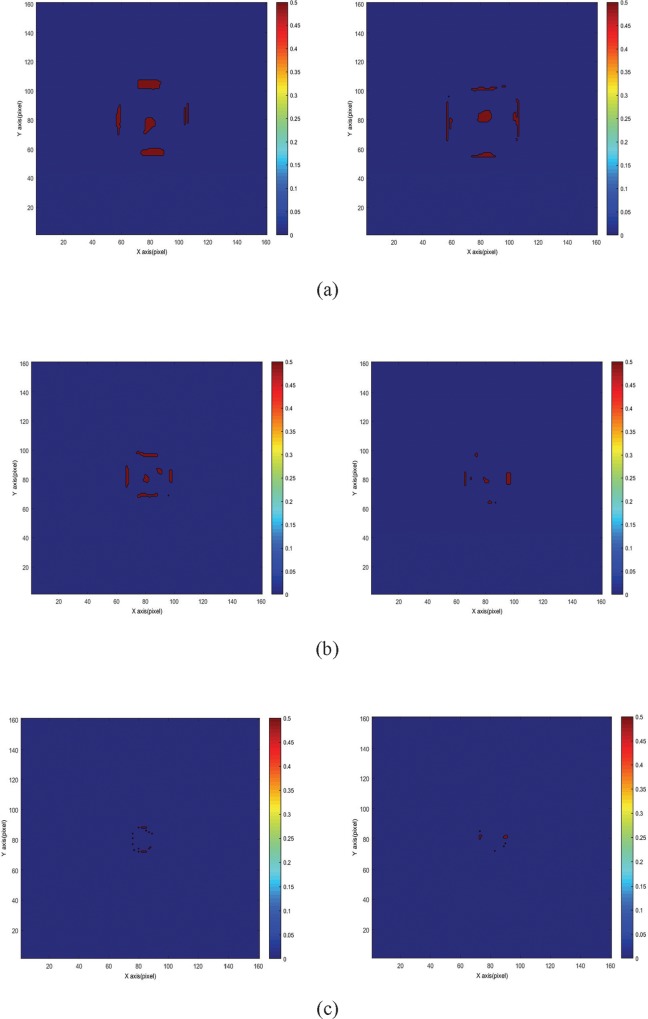
Various FS of the gamma map of NIPAM gel dosimeter at a depth of 50 mm for 24 h post-irradiation time. The gamma criteria used were DTA = 2 mm/DD = 2%. The left image represents the flattened beam, and the right image represents the FFF beam. (a) FS = 30 × 30 mm^2^, (b) FS = 20 × 20 mm^2^, and (c) FS = 10 × 10 mm^2^.

**Fig 6:**
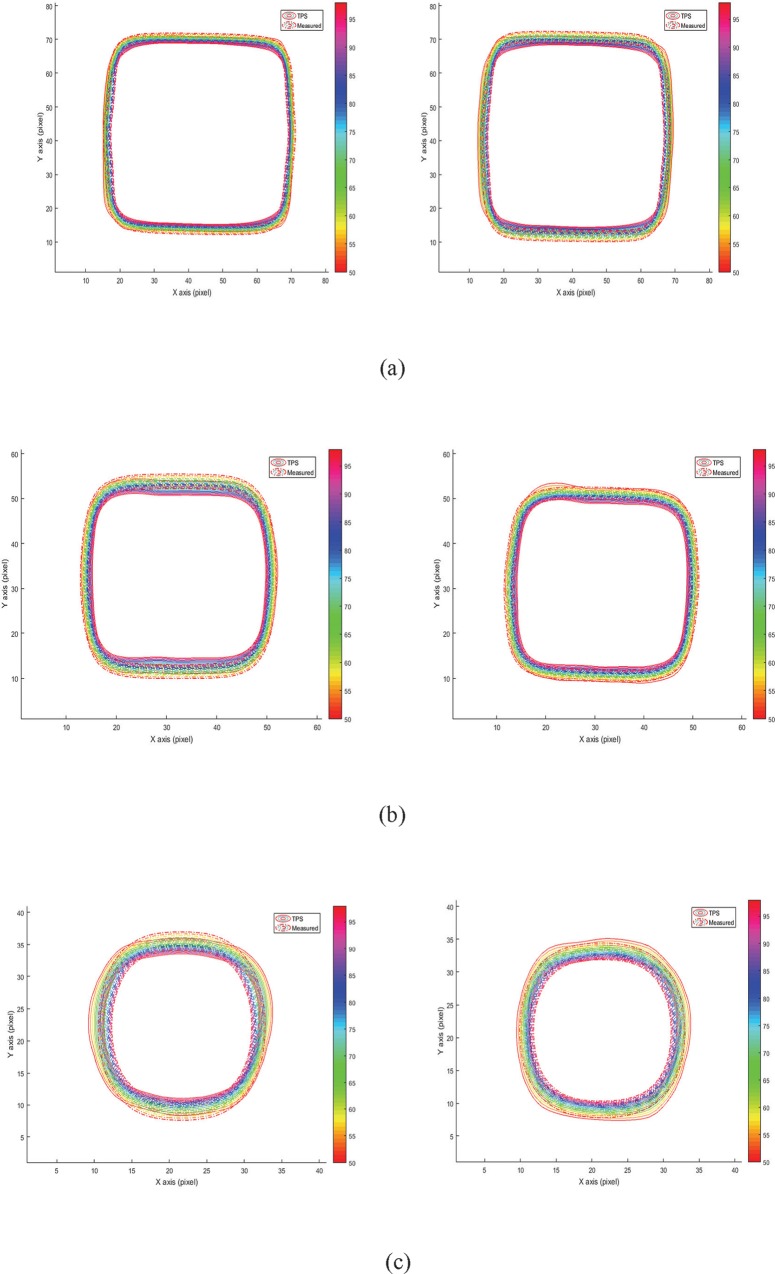
Comparison of the 100%, 90%, 80%,70%, 60%, and 50% isodose lines between the gel measurement (dotted lines) and treatment planning system (solid lines) at a depth of 50 mm for 24 h post-irradiation time. The left image represents the flattened beam, and the right image represents the FFF beam. (a) FS = 30 × 30 mm^2^, (b) FS = 20 × 20 mm^2^, and (c) FS = 10 × 10 mm^2^.

Figs 4 and 5 show the gamma maps of the flattened and FFF beams at 24 h after irradiation at a depth of 50 mm. Fig 4 displays that the rejected area, where gamma value *γ* > 1, was mostly located at the edge of the irradiation field for DTA = 3 mm/DD = 3% (Fig 6) and DTA = 2 mm/DD = 2% (Fig 5). The edge of the irradiation field is generally a high-dose gradient region, where edge enhancement may occur [33]. Furthermore, the rejected areas decreased when the FS decreased from 30 × 30 mm^2^ to 10 × 10 mm^2^ for both gamma criteria. Nevertheless, the edge enhancement effect was highly evident when the gamma criteria of 2 mm/2% were adopted (Fig 5). The FFF beam with a high dose rate (1,400 MU/min) showed rejected regions similar to those of the flattened beam with a low dose rate (600 MU/min). Therefore, the passing rates of the flattened and FFF beams presented no significant difference.

### Comparison of dose distributions and isodose lines

A comparison of the percentage isodose lines of NIPAM gel dosimeters at a depth of 50 mm and post-irradiation time of 24 h is shown in Fig 6. The dose percentages of 100%, 90%, 80%, 70%, 60%, and 50% of the gel measurement (dotted lines) and TPS (solid lines) for various FS are illustrated in the plots. The isodose maps of flattened and FFF beams were in the left and right panels of Fig 6, respectively. A comparison of the isodose lines of the TPS and the measured dose can be used to investigate the origin of dose deviations. The edge of the irradiation field is generally a high-dose gradient region and may cause edge enhancement effect in the gel dosimeter [33]. Gels are prone to induce measurement errors at high-dose gradient regions [34]. Free radical diffusion may occur near the high dose gradient regions and cause dose deviations. Fig 6 illustrates high consistency for small FS between the TPS and the measured dose at dose percentages of 50%–100%. These results showed that the edge enhancement effect in the large FS was more evident than in small FS. The isodose line showed excellent consistency between the measured data and TPS calculated data, even in high-dose gradient region near the edge of the irradiation field at FS of 10 × 10 mm^2^. Evidently, the penumbra in the flattened beam (left panel) was thicker than in the FFF beam (right panel). The edge enhancement effect in the flattened beam was also more evident than in the FFF beam. Previous research [34] also reported that filter removal can significantly reduce the amount of head scatter photons, thereby reducing doses to normal tissues and organs. Treatments can also benefit from an increased dose rate.

## Discussion

In current study, we performed the dose comparison between TPS and measured data from NIPAM gel dosimeter. In order to verify the dose accuracy of the Pinnacle3 TPS, we have conducted a commissioning procedure for Pinnacle3 TPS before experiment. As mentioned in “Irradiation and TPS” section, calibration was conducted by using the beam data obtained from small field 10 × 10 mm2 and 20 × 20 mm2 radiation, which was measured by a pinpoint ion chamber (0.015c.c) (PinPoint Ionization Chambers 30014, PTW, Germany). It ensured the comparison of the gel dosimeter and TPS was meaningful.

The passing rates between flattened and FFF beams for various irradiation FS with two gamma criteria were compared. Statistical analysis indicated no significant difference in passing rates between the flattened and FFF beams. Thus, the dose distributions irradiated by the flattened and FFF beams were the same, but the time of the FFF beam can be reduced to half of that of the flattened beam, as shown in Table 2. Therefore, the patients can reduce their unsatisfactory experiences during radiation therapy. Table 2 shows that the dose rate of FFF beam was 2.33 times higher than that of the flattened beam. Initially, we considered that severe edge enhancement may be observed in the FFF beam because high dose rate may induce fast generation of free radicals. However, the edge enhancement effect on the flattened beam was more evident than on the FFF beam. Mcauley [33] proposed simple free-radical solution polymerization systems, which indicated that when new radicals are not generated, the rate of polymerization quickly decreases to zero, because no free radicals exist. The time of the FFF beam was only half of that of the flattened beam in the current study. The similar results can be found in previous research [5,6]. Therefore, the polymerization time for FFF beam is much shorter than that of the flattened beam, thereby causing a small amount of excess polymer generated near the edge. In previous research, Olding et al. [8] claimed that the most disagreement of dose profile was located at the edge of high dose region. Moreover, limited polymerization time reduces the diffusion time of polymer. This condition results in less edge enhancement for the FFF beam. Previous results [22] also suggested that the amount of additional polymer near the edge increases with increased post-irradiation time. The same trend can be observed on the change in passing rates with post-irradiation time, as shown in Table 4 and Fig 3.

## Conclusion

Quantitative dosimetric comparison of FF and FFF radiation therapy was conducted using gamma evaluation. Three batches of NIPAM gel dosimeters for two irradiation modes (flattened and FFF beams) and three FS (30 × 30, 20 × 20, and 10 × 10 mm^2^) were prepared. Eighteen batches of NIPAM gel dosimeters were analyzed. The gamma passing rates at four depths and four post-irradiation time periods were calculated. The overall means and standard deviations of gamma passing rates with DTA = 3 mm/DD = 3% for flattened and FFF beams were 96.48 ± 2.29 and 96.62 ± 2.35, 97.97 ± 1.90 and 97.89 ± 1.28, and 100 ± 0.0 and 100 ± 0.0 for FS of 30 × 30, 20 × 20, and 10 × 10 mm^2^, respectively. In addition, the overall means and standard deviations of gamma passing rates with DTA = 2 mm/DD = 2% for flattened and FFF beams were 85.11 ± 3.45 and 86.14 ± 4.85, 89.59 ± 4.11 and 88.88 ± 3.82, and 94.94 ± 2.12 and 94.73 ± 2.14 for FS of 30 × 30, 20 × 20, and 10 × 10 mm^2^, respectively. The paired sample t-test for the two irradiation modes showed no significant difference between the flattened and FFF beams at 6 MV energy. The gamma maps illustrated that low passing rates were obtained from the edge of the irradiation regions, where high-dose gradient was located. The present results revealed that the 3D NIPAM gel dosimeter can be used for dose verification of small FS for radiation therapy with high dose rate. Therefore, the 3D NIPAM gel dosimeter can be potentially used for clinical dose verification of modern radiation technologies.
